# Supplementary figures and images for: Glucuronoxylomannan intranasal challenge prior to Cryptococcus neoformans pulmonary infection enhances cerebral cryptococcosis in rodents
Source: PLoS Pathog. 2023 Apr 28;19(4):e1010941. doi: 10.1371/journal.ppat.1010941 (PMC10171644; doi:10.1371/journal.ppat.1010941)

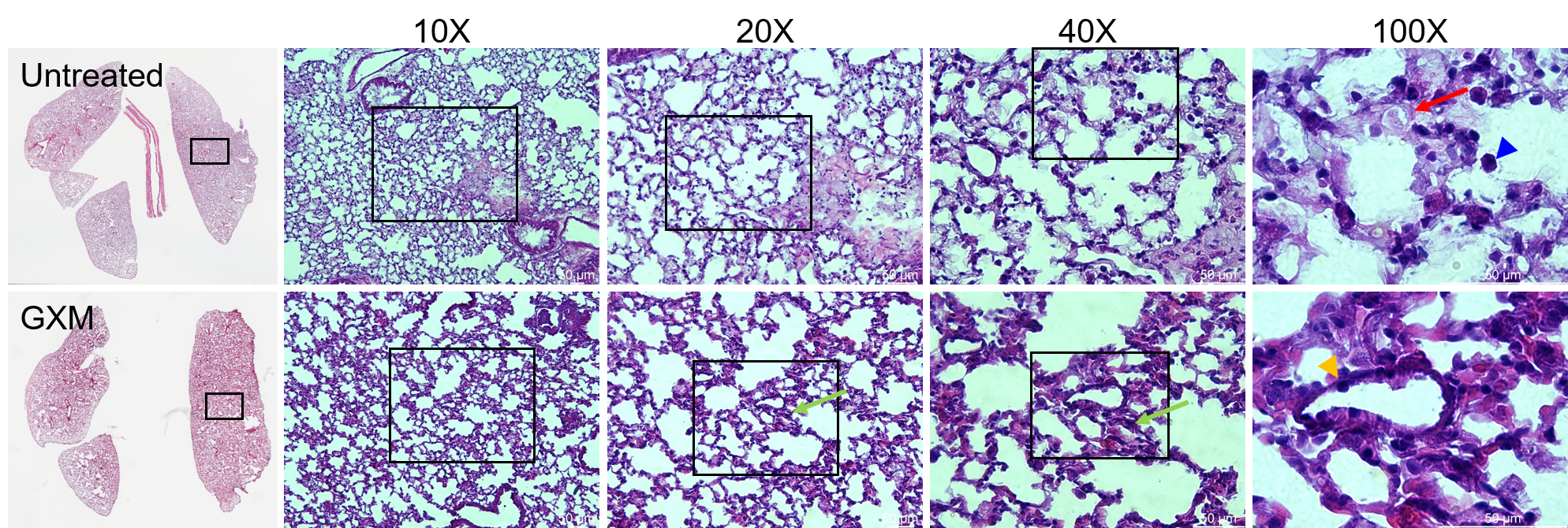

Supplement: S1 Fig — Representative lung sections (scale bar in 10-100X magnifications: 50 μm) excised from untreated (upper panels) and GXM (125 μg/mL; lower panels)-treated mice, stained with H&E, and sequentially examined under light microscopy are shown. Rectangles in the lobe, 10, 20, and 40X images indicate the lung region magnified in the following picture (on the right). (TIF) [file ppat.1010941.s001.tif]

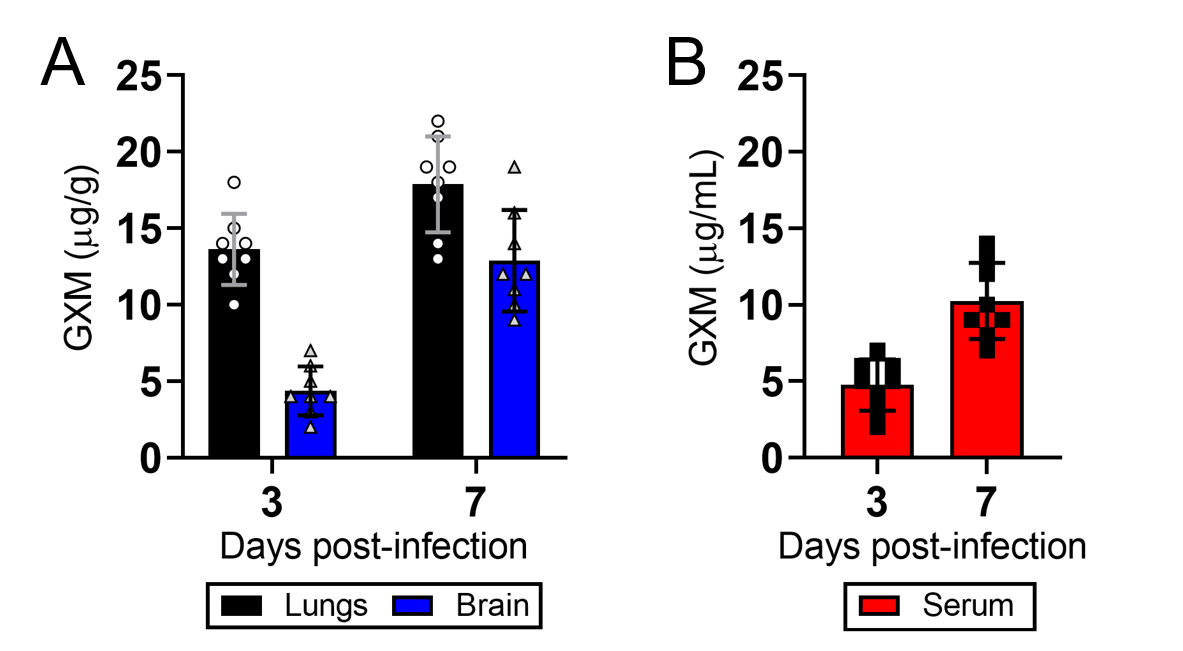

Supplement: S2 Fig — Bars and error bars denote the mean value and SDs, respectively. Each symbol represents the value for 1 mouse (n = 8 per group). (TIF) [file ppat.1010941.s002.tif]

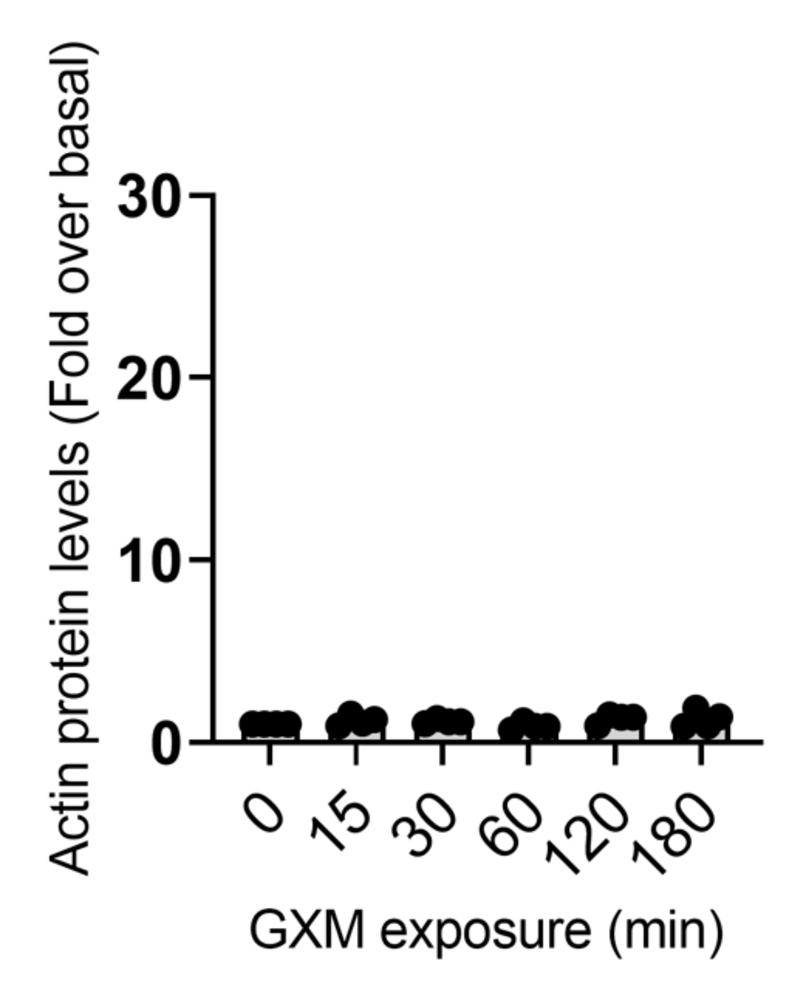

Supplement: S3 Fig — Quantitative measurements of individual band intensity in WB analysis shown in Fig 5A for (B) actin protein levels using LiCOR Image Studio software. Bars represent the means of 3 independent gel results (black circles) and error bars indicate SDs. P-value significance (P < 0.05) was calculated using one-way ANOVA and adjusted using the Tukey’s post-hoc analysis. No statistical difference was found. (TIF) [file ppat.1010941.s003.tif]

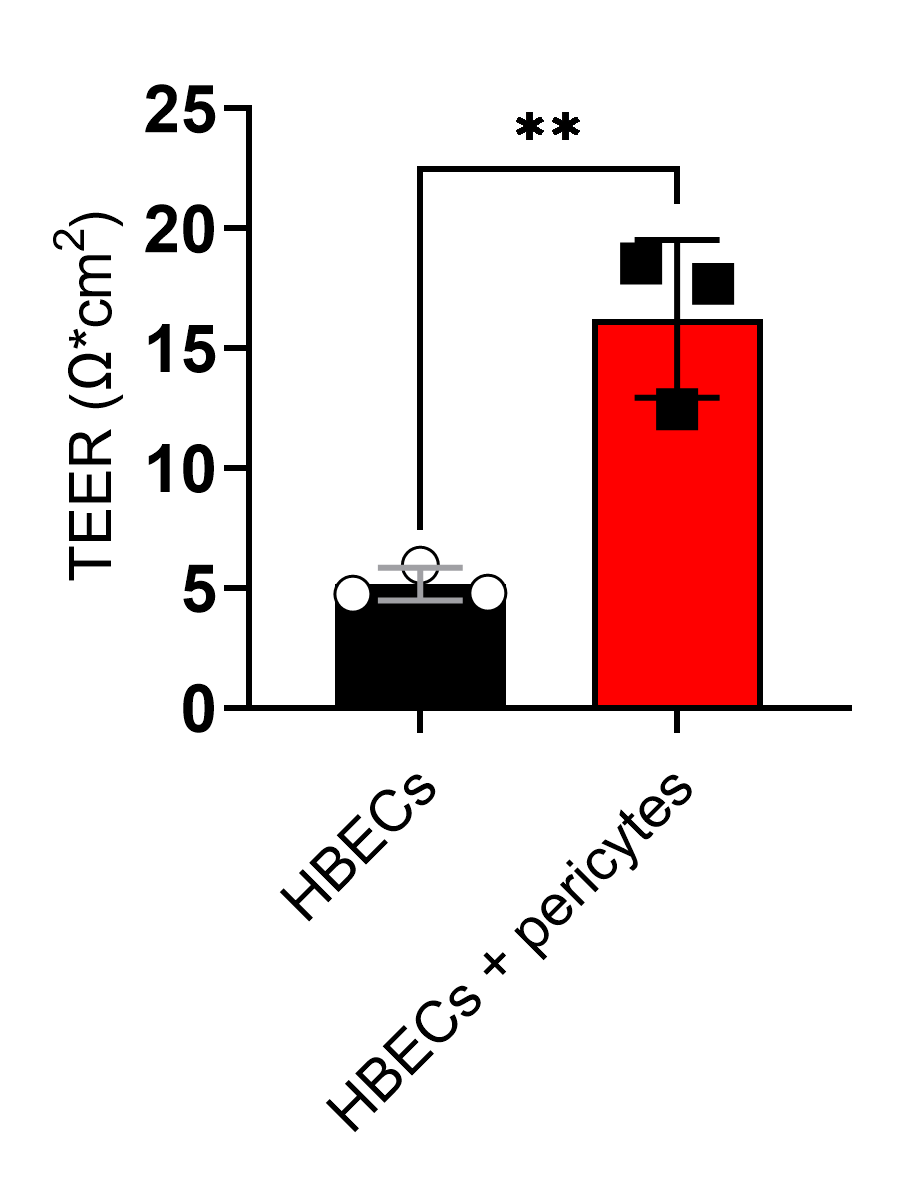

Supplement: S4 Fig — HBECs grown in 0.4 μm pore transwell inserts were placed into wells containing media alone (HBECs) or wells containing human primary pericytes (HBECs + pericytes). Bars and error bars denote the means and SDs, respectively. Each symbol denotes a single well measurement (n = 3 wells per group). Asterisks indicate P-value significance (** P < 0.01) calculated using student’s t-test analysis. (TIF) [file ppat.1010941.s004.tif]

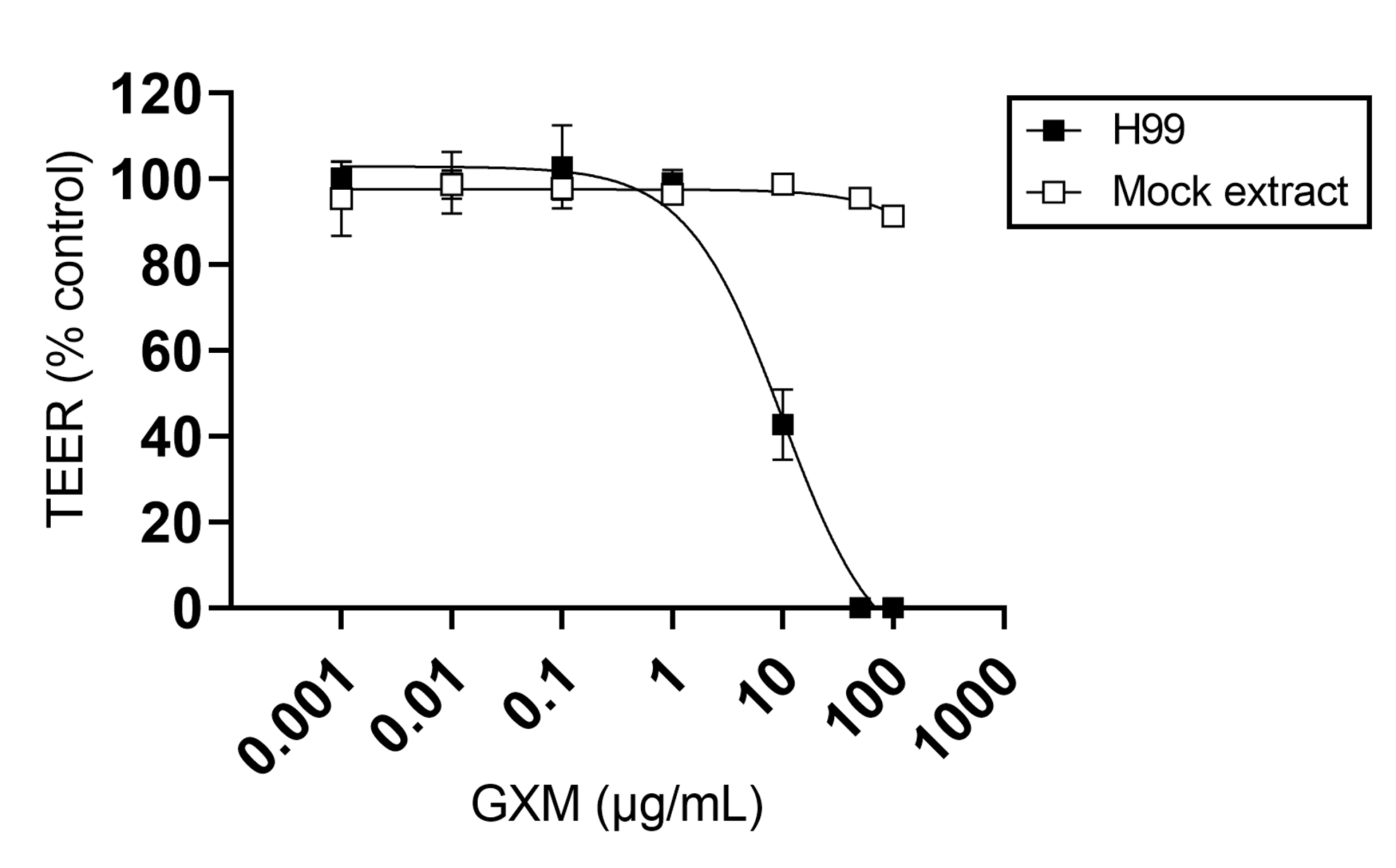

Supplement: S5 Fig — Dose-response curve: Relative TEER of HBECs incubated with purified GXM (0.001, 0.01, 1, 10, 50, or 100 μg/mL) for 2 h at 37°C and 5% CO2 was performed. HBECs were treated with a mock extract from acapsular strain cap59 and used as a negative control. Data points are averages of three different TEER measurements (n = 3 wells per group per experiment) and error bars denote SDs. (TIF) [file ppat.1010941.s005.tif]
